# Enhancing Physicochemical and Piezoelectric Properties of Eggshell Membrane Proteins by Ultrasonic-Assisted Enzymes for Food and Sensor Applications

**DOI:** 10.3390/ijms26052190

**Published:** 2025-02-28

**Authors:** Xinhua Liang, Honglian Cong, Gaoming Jiang, Haijun He

**Affiliations:** Engineering Research Center for Knitting Technology, Ministry of Education, Jiangnan University, Wuxi 214122, China; liangxh18861852560@163.com (X.L.); cong-wkrc@163.com (H.C.); jgm@jiangnan.edu.cn (G.J.)

**Keywords:** eggshell protein, response surface methodology, motion detection

## Abstract

This research sought to explore the impact of ultrasonic pretreatment on the physicochemical characteristics of proteins derived from eggshell membranes through enzymatic extraction. Response surface methodology (RSM) and Box-Behnken design were employed to identify the ideal conditions for the extraction process. The optimal parameters determined were enzyme usage at 4.2%, pH level at 2.4, a solid-to-solvent ratio of 1:20 g/mL, and an extraction time of 21.5 h. The eggshell membrane was pretreated by ultrasound before pepsin hydrolysis under optimized conditions. The findings indicated that the hydrolyzed products subjected to ultrasonic pretreatment exhibited enhanced solubility, surface hydrophobicity, water and oil retention, foaming characteristics, and emulsifying ability compared to the untreated hydrolyzed products. Furthermore, the piezoelectric properties of the protein with ultrasonic pretreatment were also significantly improved. Additionally, the protein-based piezoelectric device displayed excellent sensing performance and was successfully applied for human motion detection and precise identification of different pressure positions. These findings indicate that ultrasound has great potential to improve the physicochemical quality of eggshell membrane proteins, providing a theoretical basis and research approach for food protein modification and the preparation of green electronic devices.

## 1. Introduction

With the continuous growth of the world population, there is an increase in the demand for food, especially protein. Given protein’s function in nutrition, health products, and pharmaceuticals, the demand for protein foods is predicted to increase by 50% [1]. Animal-derived protein has good digestibility and nutritional quality. It provides a well-balanced composition of vital amino acids and serves as an emulsifier, foaming agent, and gelling agent in the food industry due to its advantageous functional properties [2]. Eggshell membrane (ESM), a prevalent food byproduct with a thickness of approximately 100 μm, is located between the egg white and the inner surface of the eggshell. It comprises 80–85% proteins, including 10% collagen (types I, V, and X), with the rest consisting of glycoproteins and lysine-derived crosslinks. Moreover, ESM includes polysaccharides like hyaluronic acid and chondroitin sulfate [3]. The highly collagenous fibers impart piezoelectricity to the ESM. More precisely, type I collagen fibers display shear piezoelectric properties as a result of the N-terminal and C-terminal telopeptides and the C6 symmetry in their crystalline chains [4]. Additionally, the closely compact and well-oriented peptide fibers create a porosity that facilitates displacement generation in ESM when subjected to external forces, thereby leading to superior piezoelectric properties [5].

Despite being a valuable source of bioactive compounds, ESM is often discarded as waste owing to its unappealing appearance and inferior quality. Inadequate disposal not only leads to potential revenue loss but also contributes to significant environmental degradation [6]. Therefore, it is necessary to use them effectively to improve waste management and relieve ecological burdens. However, the direct application of ESM is limited due to its complex structure and poor solubility. Through purification, the active ingredients can be separated from the ESM to improve their purity and biological activity so as to expand its application in food, medical health, and sensing [7].

Conventional techniques for generating protein hydrolysates and peptides involve both chemical and enzymatic hydrolysis. Chemical reagents are capable of disrupting disulfide bonds, thus enhancing protein solubility [8]. However, this approach requires many solvents and is not environmentally friendly. By contrast, the enzymatic hydrolysis method is a mature protein extraction method that improves protein extraction efficiency by increasing yields, modifying structure, and simplifying processing operations [9]. Its widespread use is attributed to its high substrate specificity and mild reaction conditions; however, its efficiency is limited by the reduced contact frequency between the enzyme and substrate [10]. Consequently, it is essential to investigate more effective methods to enhance extraction efficiency and sustainability.

In recent years, ultrasonic technology used for pretreatment before enzymatic hydrolysis has received extensive attention due to its economic feasibility, energy efficiency, high mass transfer rate, and ability to maintain the natural state of foods [11]. During the ultrasonic treatment, the shearing forces and thermal effects resulting from bubble formation and cavitation can induce structural modifications in proteins and cleave covalent bonds, thereby enhancing solubility and facilitating enzymatic access to the hydrolysis site [12]. Thus, the combined use of enzymatic hydrolysis and ultrasonic technology enhances the production of protein hydrolysates and peptides. In this context, response surface methodology (RSM) serves as a robust statistical approach to optimize intricate processes by methodically evaluating the impact of independent variables on dependent outcomes and their interactions [13].

To the extent of our knowledge, limited research has focused on enhancing protein extraction from ESM through ultrasonic-assisted techniques. This investigation examined the influence of enzyme usage, pH level, solid-to-solvent ratio, and time of enzymatic hydrolysis using a Box-Behnken design (BBD) to determine the optimal conditions for enzymatic hydrolysis. The extraction yield, protein concentration, structural characteristics, thermal attributes, and morphological features of the protein extracted under optimal conditions with and without ultrasonic assistance were compared. In the end, to improve the mechanical properties and maintain its biocompatibility for wearable sensor applications, we propose a promising approach of blending the extracted protein with biocompatible polymers. This study is of great significance for gaining insight into the structure and functionalities of ESM-based protein hydrolysates and promoting the potential utilization of ESM in multifunctional applications.

## 2. Results and Discussion

### 2.1. Statistical Analysis and Model Fitting

RSM was utilized to enhance the ultrasonic-assisted extraction of protein from ESM through a BBD, examining the impact of enzyme usage (A), pH (B), solid-to-solvent ratio (C), and extraction time (D) on the yield. The design of the experiment and the outcomes are detailed in Table S1. Each factor was evaluated at three distinct levels: (−1), (0), and (+1), with a total of 29 experimental trials, including four central points. The extraction yield of all the experiments varied from 16.21–35.52% under different processes. The analysis of the multiple linear regression equations yielded a quadratic predictive model. The resulting quadratic regression equation, identified as the optimal model, is presented as follows:

Y = 33.47 + 4.70A + 5.07B − 1.12C + 2.52D − 2.32AB − 2.34AC + 1.31AD − 0.51BC − 0.22BD − 0.05CD − 4.11A^2^ − 1.92B^2^ − 6.14C^2^ − 5.41D^2^ (1)

An analysis of variance (ANOVA) was utilized to assess the validity of the proposed statistical models. The confirmation of model coefficients through high F-values and low *p*-values (<0.05) signifies the statistical significance of the model terms. As presented in Table S2, A, B, C^2^, and D^2^ exerted an exceptionally significant influence (*p* < 0.0001) on the extraction yield of ESM, whereas factors D, AB, A^2^, and B^2^ demonstrated a significant effect (*p* < 0.05). The fitted model displayed a low *p*-value (*p* < 0.0001) and a high F-value of 16.63, indicating the excellent significance of the model. Furthermore, the favorable values (close to 1) of three robustness indicators (R^2^, Adjusted R^2^, and Predicted R^2^) and non-significant lack of fit indicate the high capability of the model in predicting the response data. The CV value of 5.28% was relatively low and acceptable, verifying the credible precision and reliability of the experimental data.

### 2.2. Optimization of the Extraction Efficiency

**Figure 1:**
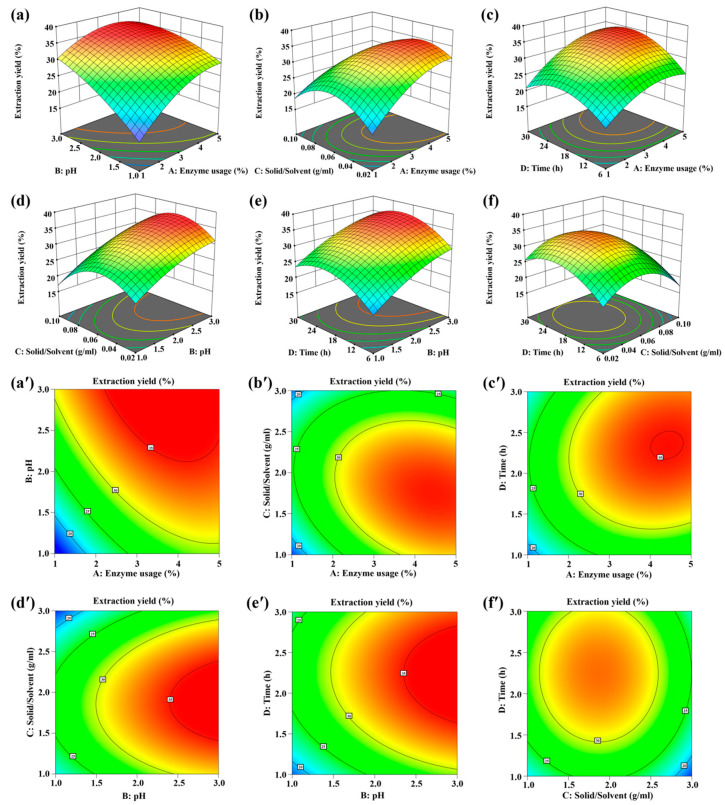
The three-dimensional (3D) response surface graphs and two-dimensional (2D) contour plots exhibiting the interactive effects of enzyme usage (**a**); pH (**b**); solid-to-solvent ratio (**c**); and time (**d**) on EY. Interaction between (**a**,**a′**) enzyme usage and pH; (**b**,**b′**) enzyme usage and solid-to-solvent ratio; (**c**,**c′**) enzyme usage and time; (**d**,**d′**) pH and solid-to-solvent ratio; (**e**,**e′**) pH and time; (**f**,**f′**) solid-to-solvent ratio and time.

Three-dimensional (3D) response surface graphs and two-dimensional (2D) contour plots, derived from the regression model, were constructed to enhance comprehension of the interactive effects among independent variables. The relationship between the two factors exhibits a positive correlation with the gradient of the 3D surface graph. Therefore, the steeper distribution of the 3D surface graph indicates a stronger correlation between the two factors. Contour plots resembling circles suggest insignificant effects of the two interactions on the response value, whereas oval contour plots indicate significant effects on the response value [14,15]. As depicted in Figure 1, the pronounced gradient of the response surface (Figure 1a) and its associated elliptical contour plot (Figure 1a′) indicate a substantial impact of the interaction between enzyme usage and pH on the extraction yield of ESM protein. In contrast, the gently sloping profiles observed in other response surfaces (Figure 1b–f), along with their relatively circular contour plots (Figure 1b′–f′), suggest that interactions among these other variables do not significantly influence the extraction yield of ESM protein. The results are also verified in Table S2 with the *p* value of AB less than 0.05.

### 2.3. Model Validation and Optimization

Through the experiment analysis of the results, the optimal extraction conditions for ESM protein were determined as follows: enzyme usage, 4.2%; pH, 2.4; solid-to-solvent ratio, 1:20 g/mL; time, 21.5 h. Under these ideal conditions, a theoretical extraction yield of 36.35% is achievable. To assess the model’s predictive accuracy, triplicate experiments were performed under the optimized conditions, yielding an actual extraction rate of 35.93%. This result exhibited no significant deviation from the theoretical value, thereby confirming the model’s robust predictive and analytical capabilities.

### 2.4. Functional Properties

**Figure 2:**
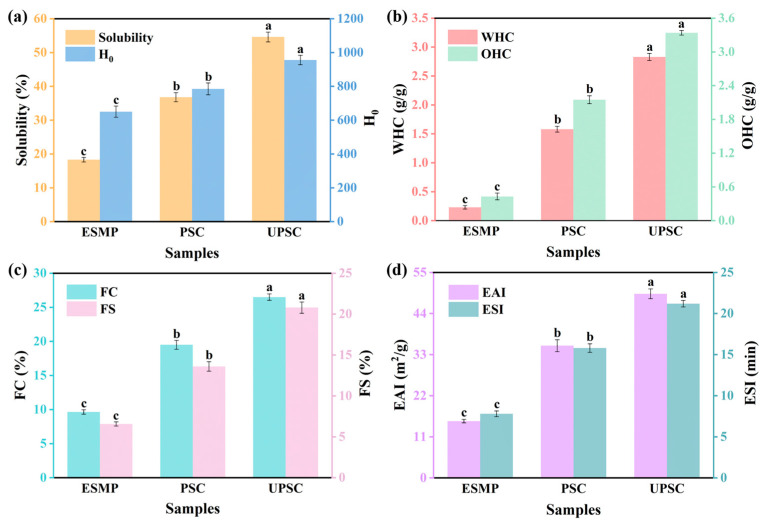
The functional properties of different samples. (**a**) solubility and H_0_; (**b**) WHC and OHC; (**c**) FC and FS; (**d**) EAI and ESI. Mean value ± standard deviations of three independent experiments were shown (*n* = 3). Different letters labeled mean significant difference (*p* < 0.05).

Solubility, a crucial attribute of proteins, influences their functional characteristics and, consequently, their practical applications [16]. As displayed in Figure 2a, the solubility of the sample after enzymatic hydrolysis was increased from 18.32% (control) to 36.83%. This could be attributed to the enzymatic capability to cleave peptide bonds within protein molecules, thereby loosening their structure [17]. Following ultrasonic treatment, the solubility of the sample was enhanced to 54.6%. The mechanical vibrations induced by ultrasound can augment the interaction surface between proteins and solvents, facilitating greater protein dissolution. Furthermore, the cavitation induced by ultrasound can disrupt the structure of protein molecules, breaking them down into smaller fragments. The localized high-temperature and high-pressure conditions resulting from cavitation also aid in dismantling the aggregated state of proteins, thereby enhancing their solubility [18].

H_0_ can reflect the exposure degree of hydrophobic amino acids on the protein surface, which can be used to assess changes in protein conformation [19]. Figure 2a shows the H_0_ of different samples. Compared with ESMP, the H_0_ of PSC and UPSC increased significantly, which was 1.21 and 1.47 times that of the control, respectively. During enzymolysis, protein molecules were cut into smaller fragments, exposing hydrophobic groups and forming more hydrophobic regions on the protein surface [20]. Furthermore, ultrasonic treatment led to greater protein molecule unfolding and increased exposure of hydrophobic residues, which interact with water molecules to expand the contact area between the protein molecules and the solvent, thereby enhancing the H_0_ of the protein [21].

WHC and OHC are two important indexes of food properties. WHC denotes the capacity of proteins to hold water, which can indicate their viscosity and texture [22]. As shown in Figure 2b, the WHC of PSC and UPSC are 1.58 and 2.15 g/g, respectively. The mechanical action of ultrasound destroys the structures of proteins and increases the surface area. The increased exposure of hydrophobic regions enhances the interaction surface between proteins and water molecules, thereby improving the capacity to retain more water molecules and leading to an elevated WHC [23]. OHC represents the protein’s capacity to bind to oil, which reflects emulsifying and thickening properties [24]. The OHC of UPSC is higher than that of PSC (Figure 2b). Ultrasonic treatment can disrupt the protein structure, facilitating greater oil penetration into the protein and thereby increasing the contact area between them. The cavitation resulting from ultrasonic treatment exposes hydrophobic groups and non-polar side chains that have a strong affinity for oil molecules, enabling proteins to more effectively adsorb and retain oils, thus enhancing the OHC [25]. Similar results were also reported in the literature [26].

FC refers to the ability of proteins to unfold and dissolve rapidly, forming a cohesive layer that surrounds the bubbles. Simultaneously, FS demonstrates the capability to generate a stable foam through the formation of a continuous intermolecular polymer network that encapsulates the air cells [27]. The FC of the protein subjected to ultrasonic treatment is 1.44 times greater than that of the untreated protein (Figure 2c). Ultrasonic treatment can cause the protein structure to extend, revealing more hydrophobic groups on the protein molecule’s surface. This enhances the adsorption and diffusion capabilities of protein molecules at the air-water interface, thereby improving their foaming properties [28]. Additionally, ultrasonic treatment can cut protein molecules into smaller particles through high shear force to improve the protein solubility, thereby improving protein’s foaming ability [29]. Furthermore, the FS of SPC after ultrasonic treatment is also enhanced (Figure 2c). The exposed hydrophobic residues caused by ultrasonic treatment are conducive to forming a more stable film at the gas-liquid interface through protein-protein hydrophobic interactions [30]. Furthermore, the combination of acid and ultrasound can enhance the viscoelastic properties of the interfacial film, inhibiting bubble coalescence, drainage, and disproportionation, thereby increasing foam stability [31].

The emulsification characteristic of proteins is a crucial factor in processed food production, typically assessed through EAI and ESI. EAI quantifies the protein’s capacity to adsorb at the oil-water interface, while ESI evaluates the maintenance of two-phase stability during emulsion creation [32]. As demonstrated in Figure 2d, ultrasonic treatment markedly improves the protein’s capability to form and stabilize emulsions. In comparison to untreated proteins, the EAI and ESI of proteins subjected to ultrasound treatment increased by 39.27% and 34.18%, respectively. The exposure of hydrophobic groups enhances the proteins’ adsorption and emulsification activity at the oil-water interface [33]. Additionally, ultrasonic shearing disrupts the molecular structure of proteins and diminishes the particle size of protein emulsions. The exposed hydrophobic regions exhibit significant adsorption capability at the oil-water boundary, facilitating the formation of a stable protein film at this interface and thereby enhancing emulsion stability [34].

Enhancing the functional attributes of enzyme-extracted ESM protein through ultrasonic treatment offers an improved material foundation and robust support for its utilization in diverse multi-functional areas.

### 2.5. Structural Properties

**Figure 3:**
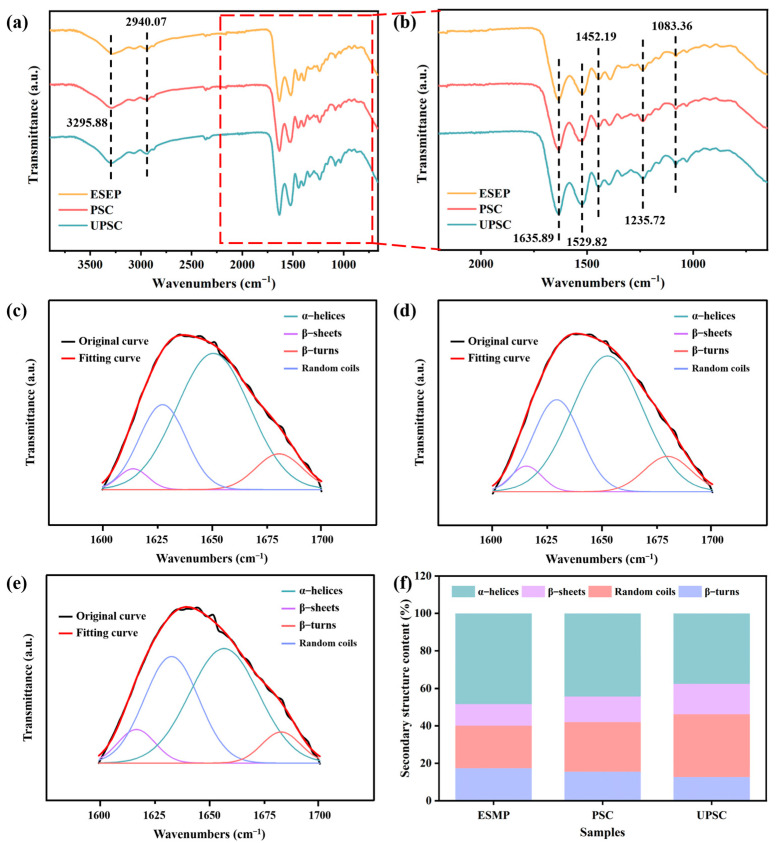
(**a**) FTIR spectra of ESMP, PSC, and UPSC; (**b**) FTIR of the range of 650 to 2200 cm^−1^ for the samples; (**c**–**e**) FTIR amide I regions of ESMP, PSC, UPSC, and deconvolution of amide I bands into individual peaks; (**f**) The secondary structure contents of ESMP, PSC, and UPSC.

FTIR spectroscopy is frequently employed to examine conformational alterations in proteins. The FTIR spectra of various samples are presented in Figure 3a,b. The general spectral profiles of all samples exhibit similarity, suggesting comparable structures and chemical constituents. The peak at 3295.88 cm⁻^1^ arises from N-H stretching vibrations associated with hydrogen bonds [35]. The band at 2940.07 cm⁻^1^ corresponds to the stretching vibrations of N-H and C-H bonds. The amide I band is detected at 1635.89 cm⁻^1^, attributable to the stretching vibrations of C=O groups that constitute the hydrogen bond chain in the three helices [36], signifying the triple helix structure of collagen. The existence of amide II is confirmed by the peak at 1529.82 cm⁻^1^, resulting from C-N stretching in conjunction with N-H bending. The absorption peak at 1452.19 cm⁻^1^ signifies the cis-configuration of the peptide bond [37], suggesting that collagen is rich in proline and hydroxyproline. The band of amide III is found at 1235.72 cm^−1^, which comes from N-H deformation and C-N stretching. The peak at 1083.36 cm⁻^1^ indicates the presence of C-O stretching or bending vibration [38]. The findings collectively demonstrate the effective extraction of collagen.

The proteins exhibit four types of secondary structures: α-helices (1650–1662 cm⁻^1^), β-sheets (1615–1637 cm⁻^1^), β-turns (1660–1690 cm⁻^1^), and random coils (1642–1655 cm⁻^1^) [39]. The amide I (1700–1600 cm^−1^) band is deconvolved using Origin 2021 software (Figure 3c–e). The secondary structure compositions of the various samples are presented in Figure 3f. Compared with ESMP, the α-helices content of PSC and UPSC decreases from 35.45 to 30.24 and 27.32%, respectively, and the β-turns content decreases from 25.68 to 23.55 and 20.36%, respectively. The β-sheets content of PSC and UPSC increases from 24.34 to 27.72 and 30.56%, respectively, and the random coils content increases from 14.53 to 18.49 and 21.76%, respectively. Proteases can specifically cut peptide bonds in proteins, thereby disrupting the stable structure of the α-helices, which becomes a stretched polypeptide chain with an increase in the β-sheets [40]. Furthermore, ultrasonic treatment can disrupt the intermolecular bonds within proteins, leading to a loosening of their molecular structure and a consequent reduction in protein stability [41].

### 2.6. SH and S-S Content

Sulfhydryl groups and disulfide bonds are crucial for sustaining the structural integrity of proteins, substantially influencing their functional characteristics. The SH groups impact the formation of both intermolecular and intramolecular covalent bonds, while disulfide bonds are essential for preserving the spatial configuration of proteins through the cross-linked network they form [42]. As exhibited in Figure S5, enzymatic hydrolysis and ultrasonic treatment significantly increase the SH content and decrease the S-S content. Enzymatic hydrolysis can disrupt the protein’s structure, revealing sulfhydryl groups initially concealed within the molecule. Furthermore, this process can selectively cleave peptide bonds, leading to the breakdown of disulfide bridges that stabilize the protein’s tertiary configuration. Furthermore, the cavitation induced by ultrasonic treatment alters the internal structure of the protein, leading to increased cleavage of S-S bonds and exposure of internal -SH groups [43].

### 2.7. Microstructure

**Figure 4:**
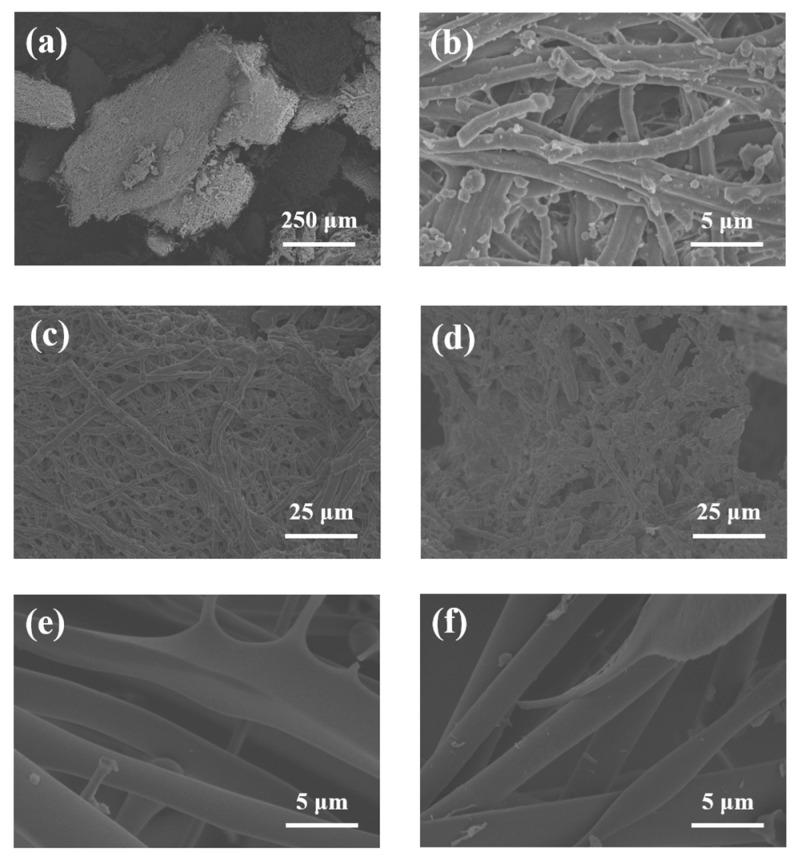
SEM image of different samples. (**a**,**b**) ESM; (**c**) ESM with enzyme treatment; (**d**) ESM with ultrasonic-assisted enzyme treatment; (**e**) PSC, and (**f**) UPSC.

The surface microstructure of various samples was examined using a scanning electron microscope. As displayed in Figure 4a,b, the surface of ESM fragments maintains the original interleaved fiber network structure, providing a large specific surface area and large interspace [44]. Figure 4c,d show the microstructure of the samples after enzyme treatment and ultrasonic-assisted enzyme treatment, respectively, and there are obvious differences between them. The enzyme dismantled the initial fiber network via hydrolysis, leading to a loosening of the network and inducing some fiber breaks [45]. Following ultrasonic treatment, the fibers shorten, the structure becomes more relaxed and fragmented, and an increased number of holes emerge on the surface. The reason may be that ultrasonic cavitation results in the expansion of some glands on the surface, which increases the contact area between the enzyme and ESM, thereby further enhancing the enzymatic properties of the protein [46]. It is found that ultrasonic treatment of ESM is more conducive to the dissolution and extraction of collagen. The structural morphology of PSC and UPSC are shown in Figure 4e,f, respectively. They both present smooth and fibrous network structures with uniform distribution, indicating that the original fiber structure of collagen is basically maintained during extraction.

### 2.8. Amino Acid Composition

The amino acid composition is a vital factor that influences protein quality and can forecast its functional attributes [47]. Table 1 illustrates the amino acid profiles of ESM, PSC, and UPSC. In ESM, the predominant amino acids are proline (11.75%), glutamic acid (11.28%), and glycine (10.81%). These components are crucial for determining the structural and functional characteristics of ESM. This result is in accordance with previous reports [48]. The main amino acid constituents of PSC and UPSC are glycine, succeeded by proline, alanine, and hydroxyproline. Collagen’s fundamental structural component is a collagen molecule comprised of three peptide chains (α-chains) that are wound into triple helical strands, distinguished by recurring (Gly-Pro-Hyp)n sequences. This configuration imparts the following features to the amino acid composition of collagen: glycine constitutes approximately one-third, while proline and hydroxyproline together comprise 20–23%, with hydroxyproline being a distinctive amino acid specific to collagen. Hydroxyproline facilitates the formation of hydrogen bonds between peptide chains, thereby preserving the stability of collagen’s triple helix structure [49]. The amino acid profiles of ASC and PSC align with those documented in the literature [50], verifying that the prepared samples consist of collagen. Furthermore, the rise in hydrophobic amino acid content following ultrasonic treatment suggests that ultrasound exposes the hydrophobic regions initially concealed within the protein structures [51], thereby enhancing the H_0_ of the proteins. The hydrophobic amino acids are crucial for sustaining protein structure and stabilizing its triple helix configuration. An increase in their concentration may further facilitate interactions among protein molecules, ultimately bolstering protein stability and enhancing its functional attributes. Moreover, changes in amino acid composition can influence protein solubility, emulsifying properties, and foaming characteristics, thus expanding its range of applications in the food industry and biological materials.

### 2.9. Mechanical Properties

**Figure 5:**
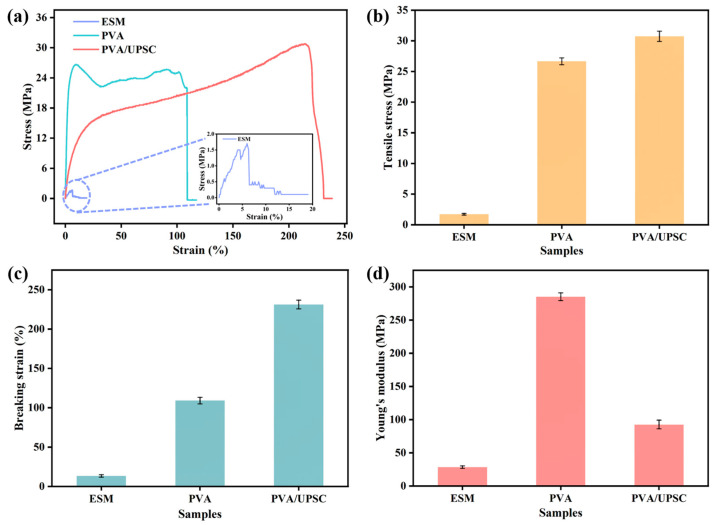
Mechanical properties of different samples. (**a**) Stress-strain curves; (**b**) The maximum tensile stress; (**c**) Breaking strain; (**d**) Young’s modulus of different samples. Mean ± SD of three independent experiments are shown (*n* = 3).

Figure S6 shows the photographs of the prepared PVA film and PVA/UPSC film, respectively. The PVA film exhibits superior transparency relative to the PVA/UPSC film, possibly attributable to the intermolecular hydrogen bonding between PVA and ESM protein. This bonding alters the molecular arrangement within the film, thereby influencing its transparency [52]. Figure 5a illustrates the stress-strain curves of different samples. ESM demonstrates inadequate mechanical characteristics because of its brittle nature [53]. The presence of ESM proteins provides sufficient flexibility for the PVA film. In the absence of UPSC, the PVA film was mechanically stiff and brittle, displaying a high Young’s modulus of 285.19 MPa, a maximum tensile stress of 26.67 MPa, and a fracture strain of 109.05% (Figure 5b–d). Under the plasticizing effect of UPSC, the Young’s modulus of the PVA/UPSC film decreased to 92.59 MPa with a high tensile stress of 30.75 MPa and a high strain of 231.16%. This may be attributed to the fact that the hydrogen bond formed by PVA and UPSC can act as a “node” for stress dispersion, thereby reducing the stress on individual molecular chain segments and helping to improve the flexibility of the film [54]. Furthermore, the formation of hydrogen bonds also enhances the bond strength between the molecular chains, which can disperse and transfer stress more effectively under external forces [55], thus increasing the tensile stress and fracture strain of the PVA/UPSC film.

### 2.10. Piezoelectric Performance

The likely mechanism underlying the enhanced piezoelectricity in PVA/UPSC can primarily be ascribed to the intrinsic dipole moment of the protein and the hydrogen bonding interactions between the protein and PVA. To verify the piezoelectric characteristics, d_33_ measurements were conducted for all samples. As shown in Figures S7 and S8, the d_33_ of PVA film is low and almost negligible due to its non-existent piezoelectricity. The ESM displays a d_33_ value of 13.4 pC/N, consistent with the value reported in the previous literature [56]. The PVA/UPSC film exhibits a high value of 21.5 pC/N, mainly due to the formation of more charge separation regions caused by the polar groups of PVA and protein, thus enhancing the piezoelectric effect.

**Figure 6:**
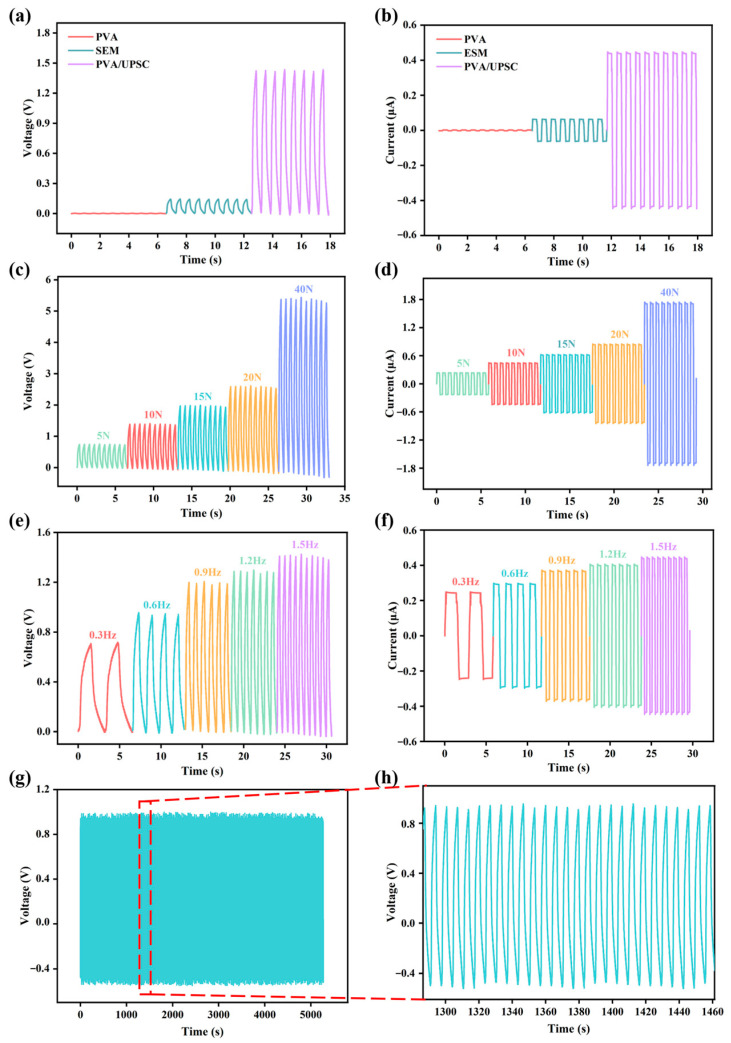
Piezoelectric performance of all samples. (**a**) The voltages and (**b**) currents of different samples; (**c**) Piezoelectric voltages and (**d**) currents at 1.5 Hz under different applied forces (5–40 N); (**e**) Piezoelectric voltages and (**f**) currents under 10 N at different frequencies (0.3–1.5 Hz); (**g**,**h**) Stability test of the device under 3000 working cycles.

Figure 6a,b demonstrate the voltage and current of different samples under 10 N with 1.5 Hz. The PVA/UPSC film exhibits significantly enhanced piezoelectric signals, which are consistent with the values of d_33_ (Figure S8). Then, a comprehensive investigation was conducted into the piezoelectric output of the PVA/UPSC film to evaluate its sensing capabilities. Figure 6c,d depict the output voltage and current generated under periodic forces ranging from 5 to 40 N. The output grows as the applied force increases, suggesting that the device can detect changes in pressure. The output performances were also conducted under different frequencies (0.3–1.5 Hz) with an external pressure of 10 N (Figure 6e,f) to evaluate the device’s ability in various environments. The device’s voltage and current increased in tandem with the rise in frequency, suggesting that a greater strain rate facilitates the buildup and subsequent release of piezoelectric charges. Furthermore, the mechanical durability of the device was measured under 10 N force and 1.5 Hz frequency (Figure 6g,h and Figure S9). Under dynamic testing for 3000 cycles, the output remained virtually stable with minimal fluctuations, highlighting its promising potential in flexible wearable electronics.

**Figure 7:**
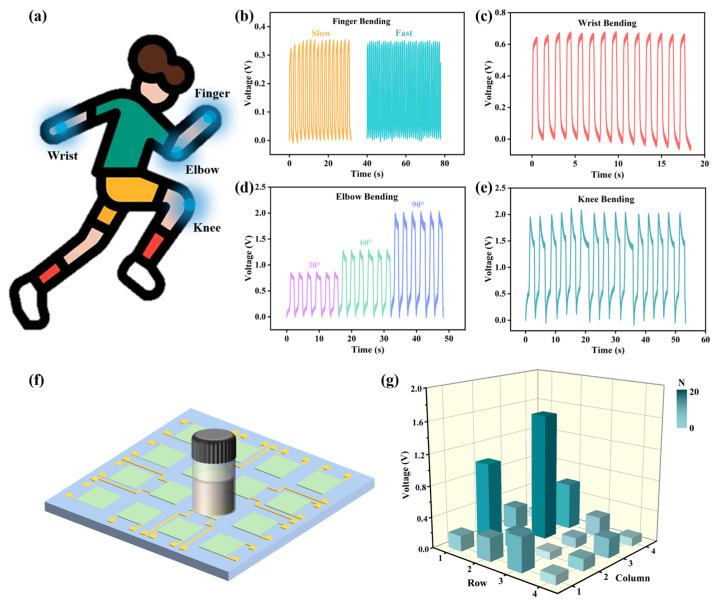
Applications of the device in detecting various human motions. (**a**) Diagram of the test on the human body; (**b**–**e**) The signals corresponding to finger bending, wrist bending, elbow bending, and knee bending; (**f**) Schematic diagram of a 4 × 4 pixel sensing array; (**g**) The voltages corresponding to spatial pressure distributions.

Based on the excellent sensing performance of the device, we further explored its practical applications in detecting human motion to verify the potential of flexibility and wearability. On this basis, the device was attached to various body parts (finger, elbow, wrist, and knee) to monitor the body movements (Figure 7a). As shown in Figure 7b, the device displays various responses under different bending frequencies, suggesting that the sensor can precisely monitor the frequency and amplitude of the finger. The waveform resulting from wrist flexion exhibits distinct patterns, with the voltage peaking at 0.63 V (Figure 7c). Figure 7d shows the voltages yielded by the movement of the elbow. As the bending angle of the elbow increases from ~30 to ~90°, the output voltages rise from 0.84 to 1.98 V, potentially owing to the enhanced force and contact area between the finger and the device resulting from the greater bending angle. Furthermore, the device is capable of detecting signals from knee bending during walking (Figure 7e). These findings indicate that ESM protein-based sensors hold promising potential for tracking the magnitude and frequency of human movement, with applications in rehabilitation and the monitoring of intelligent robot motions.

Furthermore, a sensing array with 4 × 4 pixels was assembled by 16 pieces of 1 × 1 cm PVA/UPSC films (Figure 7f). Each film sensor remains independent, and the pressure on each pixel is monitored in real time. External pressure was produced by placing reagent bottles with different water weights (10–150 g) on the array, and the voltage of each pixel was measured to map the spatial pressure distribution (Figure 7g). The voltage at the position of the serum vial was positively correlated with the pressure magnitude, indicating that the array could accurately identify the location and magnitude of external pressure. Therefore, ESM protein-based piezoelectric sensors are considered promising candidates for human-computer interaction and e-skin applications.

## 3. Materials and Methods

### 3.1. Raw Materials and Reagents

Fresh chicken eggshells were supplied by the school canteen. The ESM was extracted by manually peeling it from the eggshells, then thoroughly washed with distilled water and dried at ambient temperature until fully dry. The dried ESM was crushed into small particles (ESMP) and stored in clean packaging bags at –18 °C until use.

The pepsin (3000 U/mg) was purchased from Shanghai Merrier Biochemical Technology Co., Ltd. (Shanghai, China). Sodium hydroxide (NaOH), glacial acetic acid, and other chemicals utilized in this research were purchased from Sinopharm Group Chemical Reagent Co., Ltd. (Shanghai, China). All additional chemicals and reagents employed in the study were of analytical quality.

### 3.2. Enzymatic Extraction of Eggshell Membrane Protein

Dried ESM particles were dispersed in distilled water at different solid-to-liquid ratios, and the pH was modified using acetic acid (0.5 mol/L). Pepsin with different mass fractions (based on ESM particle) was added for enzymatic hydrolysis at 4 °C. The insoluble residue was subsequently rinsed with distilled water and dried in the oven to facilitate the subsequent determination of the ESM extraction rate.

### 3.3. Experimental Design

Figures S1–S4 illustrate the impact of enzyme usage, pH, solid-to-solvent ratio, and time on the extraction yield of ESM protein. To identify the optimal conditions for enzymatic hydrolysis, the Design–Expert 13 software was employed to construct a BBD, assessing the effects of enzyme usage (A, 1.0–5.0%), pH (B, 1–5), solid-to-solvent ratio (C, 1:10–1:50 g/mL) and time (D, 6–48 h) on protein extraction from ESM. The extraction yield (EY) served as the response variable. The experimental setup comprised 29 trials, with three repetitions at the central point, and was conducted in a random order to minimize unforeseen variations. The resulting data were analyzed using a quadratic polynomial regression model, as demonstrated:

(2) $$Y=\beta_{0}\sum_{i=1}^{n}\beta_{i}X_{i\;}+\sum_{i=1}^{n}\beta_{ii}X_{ii}^{2}+\sum_{i<j=1}^{n}\beta_{ij}X_{i}X_{j}\;\;\;\;\;\;\;$$

where *Y* denotes the predicted response variable, *X_i_* and *X_j_* signify the independent variables, *β*_0_, *β_i_*, *β_ii_*, and *β_ij_* correspond to the constant, linear, quadratic, and interaction terms, respectively, and “*n*” indicates the total number of independent variables.

### 3.4. Ultrasonic-Assisted Enzymatic Extraction (UAEE) of Eggshell Membrane Protein (ESMP)

Prior to enzymatic hydrolysis, the ESM suspension underwent treatment in an ultrasonic bath for 20 min, after which the enzymatic hydrolysis was carried out under optimal conditions. The samples without ultrasonic pretreatment were used as blank control.

### 3.5. Isolation of Pepsin Soluble Collagen (PSC) and Ultrasonic-Assisted Pepsin Soluble Collagen (UPSC)

The supernatants resulting from enzymatic hydrolysis, both with and without ultrasonic pretreatment, were gathered, and their pH was adjusted to 7~8 using a 2.5 mol/L NaOH solution. Subsequently, NaCl was added to achieve a final concentration of 4 mol/L to precipitate the supernatants, which were then stored overnight at 4 °C. Following this, centrifugation was performed at 8000 r/min for 15 min. The resultant precipitate was redissolved in a 0.5 mol/L acetic acid solution. The resulting solution underwent dialysis in acetic acid (0.1 mol/L) within a dialysis membrane featuring a molecular weight cut-off of 8–14 kDa for 48 h at 4 °C, with the solution being refreshed every 6 h. Following this, the solution was dialyzed in distilled water, with frequent water changes until a neutral pH was achieved. The dialysate that did not undergo ultrasonic pretreatment was subsequently freeze-dried and designated as PSC, while the dialysate that received ultrasonic pretreatment was also freeze-dried and labeled as UPSC.

### 3.6. Properties Characterization

#### 3.6.1. Solubility

The solubility of the protein extract was assessed following the procedure outlined by Tsumura et al. [57] with minor adjustments. A 100 mg sample was dissolved in 10 mL of phosphate buffer (0.01 mol/L, pH 7.0). This solution was then stirred for 30 min and subsequently centrifuged at 10,000× *g* for 20 min. The protein content in the resulting supernatant was quantified using the Bradford method, with bovine serum albumin serving as the reference standard [58]. Protein solubility was calculated using the following formula:

S (%) = (C_2_/C_1_) × 100 (3)

where S denotes solubility, C_1_ represents the protein concentration in the supernatant, and C_2_ corresponds to the total protein concentration in the sample.

#### 3.6.2. Surface Hydrophobicity (H_0_)

The H_0_ of the protein extract was assessed using 8-aniline-1-naphthalene sulfonate (ANS) as the fluorescence probe [59]. The sample was dissolved in a 0.01 mol/L phosphate buffer (pH 7.0) and subsequently centrifuged at 12,000× *g* for 20 min, after which the supernatant was diluted to a concentration of 0.2–1.0 mg/mL. Next, 20 μL of an 8.0 mmol/L ANS solution was added to a 4 mL aliquot of the sample, and the mixture was incubated in the dark for 5 min. The fluorescence intensity, measured at an excitation wavelength of 270 nm and emission wavelengths ranging from 400–650 nm, was assessed using a fluorescence spectrophotometer (Cary Eclipse, Varian Inc., Palo Alto, CA, USA). The initial slope of the linear regression between protein concentration and fluorescence intensity was defined as the H_0_ of the sample.

#### 3.6.3. Water Holding Capacity (WHC) and Oil Holding Capacity (OHC)

WHC was determined using the method described by Hadidi et al. with minor modifications [60]. A protein extract weighing 0.5 g (W_0_) was dispersed in 10 mL (W_1_) of distilled water at ambient temperature for 30 min, after which it was centrifuged at 5000× *g* for 20 min. The supernatant liquid was then decanted and its volume measured using a graduated cylinder (W_2_). The WHC was determined according to the following formula:

WHC (g water/g) = (W_1_ − W_2_)/W_0_ (4)

OHC of the protein extract was measured based on the method reported by Romdhane et al. with minor modifications [61]. With slight adjustments, 0.5 g of the sample (O_0_) was transferred to a centrifuge tube, mixed with 10 g (O_1_) of soybean oil, and stirred for 10 min. This was followed by centrifugation at 4000× *g* for 20 min. Subsequently, the free oil was isolated and weighed (O_2_). The OHC was calculated using the following formula:

OHC (g oil/g) = (O_1_ − O_2_)/O_0_ (5)

#### 3.6.4. Foaming Properties

The foaming capacity (FC) and foam stability (FS) of the samples were assessed according to the method of Shahidi et al. with slight modifications [62]. Specifically, 0.5 g of each sample was dispersed in 10 mL of distilled water and homogenized for 10 min using a homogenizer (D-160, Scilogex, Rocky Hill, CT, USA). The FC and FS were evaluated by measuring the total volume of the mixture initially and after 30 min of shaking, respectively. FC (%) and FS (%) were calculated as follows, respectively:

FC (%) = [(V_1_ − V_0_)/V_0_] × 100 (6)

FS (%) = (V_2_/V_0_) × 100 (7)

where V_0_ denotes the initial total volume following homogenization, V_1_ represents the total volume after homogenization, and V_2_ indicates the total volume subsequent to shaking for 30 min.

#### 3.6.5. Emulsifying Properties

The emulsifying properties were measured according to the method used by Pearce et al. with minor modifications [63]. Solutions of proteins (30 mL) at a concentration of 5% were prepared, after which 10 mL of soybean oil was incorporated and the mixture was homogenized at 5000× *g* for 5 min. Aliquots (50 μL) were withdrawn from the bottom of the container immediately and 10 min post-homogenization, then diluted with 5 mL of a 0.1% SDS (*w*/*v*) solution. The absorbance of these solutions was measured at 500 nm using a UV-Vis spectrophotometer (Lambda950, PerkinElmer, Waltham, MA, USA). The Emulsifying activity index (EAI) and Emulsion stability indices (ESI) were determined using the subsequent equations:

EAI (m^2^/g) = [(2 × 2.303 × A_0_)/VW] × 100 (8)

ESI (min) = [A_0_/(A_1_ − A_0_)] × 10 (9)

where A_0_ represents the absorbance at 500 nm determined post-emulsification, V denotes the volumetric proportion of oil, W signifies the weight of protein (g), and A_1_ indicates the absorbance following 30 min of homogenization.

### 3.7. Fourier Transform Infrared Spectroscopy (FTIR)

The FTIR spectra of the samples were obtained using the following procedure [64]. In summary, each sample was blended and pulverized with KBr in a 1:100 (g/g) ratio, after which the resulting mixture was compressed into thin discs. Subsequently, the analysis was performed using a spectrometer (Nicolet is10, Thermo Fisher Scientific, Waltham, MA, USA), conducting 32 scans within the range of 400–4000 cm⁻^1^. The analysis of the amide I region (1600–1700 cm^−1^) was conducted using PeakFit 4.12 software to assess alterations in the secondary structure of ESM proteins by quantifying the peak areas of individual sub-peak bands [65].

### 3.8. Determination of Free Sulfhydryl (SH) and Disulfide Bond (S-S) Content

The levels of free sulfhydryl groups and disulfide bonds were assessed using a modified version of the specified procedure [66]. Freeze-dried samples were reconstituted in Tris-glycine buffer (comprising 2.5% SDS, 0.086 M Tris, 0.09 M glycine, and 0.004 M EDTA at pH 8.0) to a concentration of 2 mg/mL. This solution was incubated at 25 °C for 24 h with constant agitation and subsequently centrifuged at 10,000× *g* at 4 °C for 15 min. A 0.05 mL volume of Ellman’s reagent solution was added to the collected supernatant with rapid mixing, followed by a 15-min reaction period at room temperature. The absorbance at 412 nm was subsequently measured using a UV spectrophotometer (UV2450, Shimadzu, Kyoto, Japan).

To determine the total sulfhydryl content, the protein sample was solubilized in a urea-Tris-glycine buffer (comprising 2.5% SDS, 10 M urea, 0.086 M Tris, 0.09 M glycine, and 0.004 M EDTA at pH 8.0), followed by the addition of 50 μL of β-mercaptoethanol. The resulting solution was incubated at 25 °C for 60 min and subsequently centrifuged at 10,000× *g* for 20 min. Subsequently, the supernatant obtained was dissolved in 6 mL of a 12% Trichloroacetic acid solution (*w*/*v*). The precipitate, following centrifugation, was then resuspended in 2.5 mL of Tris-glycine buffer, with the inclusion of 0.04 mL of DTNB reagent (Dithiobis-2-nitrobenzoic acid) at a concentration of 4 mg/mL, and incubated for 30 min at 25 °C. The absorbance was measured at 412 nm using a UV spectrophotometer. The free and total sulfhydryl content was determined using the provided equations:

C_SH_ (μmol/g) = (73.53 × A_412_ × D)/C (10)

where A_412_ denotes the UV absorbance of the sample at 412 nm, C represents the sample concentration, and D indicates the dilution factor, respectively.

The SS content was determined using the subsequent formula:

C_SS_ (μmol/g) = (C_SH1_ − C_SH2_)/2 (11)

where C_SH1_ is the total sulfhydryl content and C_SH2_ is the free sulfhydryl content.

### 3.9. Scanning Electron Microscopy (SEM)

The surface morphology of various samples was examined using a scanning electron microscope (su1510, Hitachi High–Technologies Corporation, Tokyo, Japan) with an accelerating voltage set at 15.0 kV. Prior to SEM analysis, the samples were affixed to the mounting platform using double-sided carbon tape and coated with gold powder to serve as a conductive medium.

### 3.10. Amino Acid Composition

The amino acid profile of the protein sample was analyzed utilizing an automated amino acid analyzer (S433D, Sykam, Fürstenfeldbruck, Germany). The sample was enclosed in a vacuum hydrolysis tube under a nitrogen atmosphere and hydrolyzed with 6 mol/L HCl at 110 °C for 24 h. Subsequently, the hydrolyzed sample was transferred to a stoppered tube, neutralized with 10 mol/L NaOH, and adjusted to a final volume of 25 mL with distilled water. The sample underwent filtration through a 0.22 μm membrane filter, followed by centrifugation at 15,000× *g* for 30 min. The resulting supernatants were then collected for the analysis of amino acid composition.

### 3.11. Preparation of PVA/UPSC Film

The PVA solutions (10% *w*/*v*) were prepared by dissolving 5 g of PVA granules in 50 mL of deionized water at 90 °C for 2 h while stirring. Once the solutions had cooled to ambient temperature, 0.1 g of UPSC was added and stirred for 1 h. Subsequently, the resulting mixture was transferred to a culture dish and dried in an oven at 50 °C for 24 h. The PVA/UPSC film was then carefully removed from the dish and stored for subsequent analysis. Pure PVA film was also prepared as blank controls.

### 3.12. Mechanical Properties

The mechanical properties of different samples (10 × 20 mm) were carried out using a universal testing machine (MTS Exceed E43, Shanghai, China) in accordance with the GB/T 1040.3-2006 standard [67]. Testing was conducted at a tensile rate of 50 mm/min, with an ambient temperature of 25 ± 2 °C and a relative humidity of 60 ± 2%. Each experiment was replicated three times.

### 3.13. Assembly of Piezoelectric Sensors

The fabricated films were divided into 2 × 2 cm^2^ sections and placed between two conductive copper strips, after which two copper wires were attached to each side of the copper strips. The device was then encapsulated with polyimide (PI) tape as a protective layer to avoid external mechanical damage and triboelectric effects.

### 3.14. Piezoelectric Properties

The output voltage and current were recorded using an electrometer (Keithley 6514, Keithley Instruments, Inc., Cleveland, OH, USA). The periodic force and frequency were controlled using a Mark-10 mechanical testing instrument.

The piezoelectric constant (d_33_) of the films was assessed using a YE2730A quasi-static d_33_ m (Lianeng Electronic Technology Co., Ltd., Jiangsu, China). Prior to the experiment, informed consent was secured from all participating volunteers, adhering to the local ethical guidelines.

### 3.15. Statistical Analysis

Each experiment was performed in triplicate, and the findings were reported as mean ± standard deviation (SD). Optimization was performed using Design Expert software (Huanzhong Ruichi Technology Co., Ltd., Beijing, China) and response surface methodology. The data were analyzed using one-way ANOVA via IBM SPSS Statistics 26, and *p* values less than 0.05 were deemed statistically significant.

## 4. Conclusions

This research comprehensively examined the effects of ultrasonic treatment on the functional and piezoelectric characteristics of ESM proteins, establishing a robust theoretical and experimental foundation for utilizing ESM proteins in food science, biomaterials, and related areas. The PVA/UPSC film exhibited a high voltage of 1.42 V, about 8.8 times that of pure ESM. When detecting human movements, the voltage generated by the sensor increased from 0.34 V for small bending (finger bending) to 1.98 V for large bending (knee bending). In addition, a 4 × 4-pixel sensor array assembled from PVA/UPSC films can accurately identify the location and weight (10–150 g) of different bottled water with a voltage range of 0.12 V to 1.6 V. While ultrasonic treatment markedly enhances the functional attributes of ESM proteins, its impact on their long-term stability warrants additional research. Several factors, such as storage conditions, light exposure, temperature variations, and possible interactions with other constituents in a formulated product, can influence long-term stability. Future studies could focus on evaluating the stability of the ESM proteins over extended periods. This could involve storing the proteins under different conditions and regularly assessing their functional and piezoelectric properties to determine any changes over time. Additionally, stabilizers or protective agents can be explored to enhance the long-term stability of the proteins. In addition, the specific application and performance optimization of ESM proteins in wearable devices are also important directions for future research. Exploring the underlying mechanisms responsible for the enhancements in functional and piezoelectric properties following ultrasonic treatment would be advantageous. Overall, this study provides a new perspective and method for the extraction and functional optimization of ESM proteins and lays a solid foundation for applying ESM proteins in multifunctional fields. Future research should focus on the stability of ESM proteins and specific applications in wearable devices to utilize ESM proteins in practical applications.

## Figures and Tables

**Table 1:** Amino acid composition of different samples.

Amino Acid	ESMP	PSC	UPSC
Aspartic acid (Asp)	8.53	5.43	5.15
Threonine (Thr)	6.85	2.52	2.16
Serine (Ser)	9.33	4.61	4.23
Glutamic acid (Glu)	11.28	5.84	5.35
Glycine (Gly)	10.81	25.85	26.42
Alanine (Ala)	4.35	11.94	12.14
Valine (Val)	7.47	2.63	2.74
Methionine (Met)	2.51	1.21	1.03
Isoleucine (lle)	3.35	1.52	1.61
Leucine (Leu)	5.22	2.61	2.67
Tyrosine (Tyr)	2.13	0.86	0.71
Phenylalanine (Phe)	1.52	1.03	1.12
Histidine (His)	4.25	1.23	1.02
Lysine (Lys)	3.41	2.25	2.04
Arginine (Arg)	5.82	8.06	8.23
Proline (Pro)	11.75	12.56	13.17
Hydroxyproline (Hyp)	1.42	9.85	10.21

## Data Availability

The data presented in this study are available on request from the corresponding author.

## Supplementary Materials

The supporting information can be downloaded at: https://www.mdpi.com/article/10.3390/ijms26052190/s1.
