# Supplementary material for: Enhancing Physicochemical and Piezoelectric Properties of Eggshell Membrane Proteins by Ultrasonic-Assisted Enzymes for Food and Sensor Applications
Source: Int J Mol Sci. 2025 Feb 28;26(5):2190. doi: 10.3390/ijms26052190 (PMC11901099; doi:10.3390/ijms26052190)
Supplement: Supplementary file 1 [file ijms-26-02190-s001.zip › ijms-3439374-supplementary.pdf]

Supporting Information

Enhancing physicochemical and piezoelectric properties of eggshell membrane proteins by ultrasonic-assisted enzymes for food and sensor applications

Xinhua Liang [1], Honglian Cong [1], Gaoming Jiang [1], and Haijun He [1,*]

[1]Engineering Research Center for Knitting Technology, Ministry of Education, Jiangnan University, Wuxi, Jiangsu 214122, China;

liangxh18861852560@163.com (X.H.L.); cong-wkrc@163.com (H.L.C.); jgm@jiangnan.edu.cn (G.M.J.); hhj@jiangnan.edu.cn (H.J.H.)

[*]Correspondence: hhj@jiangnan.edu.cn

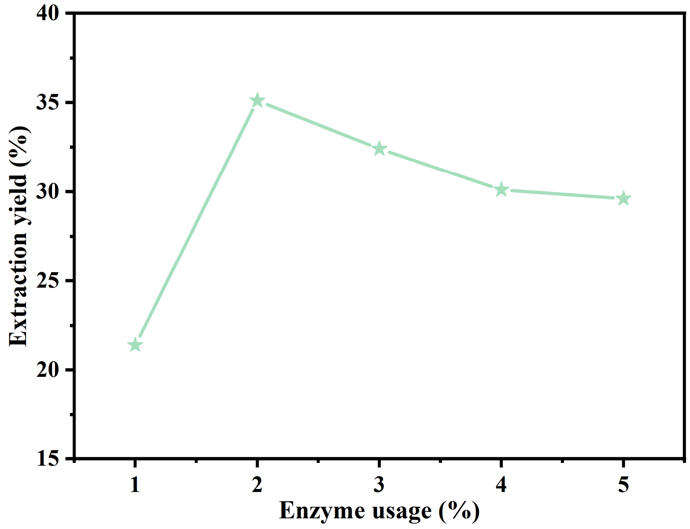

Figure S1. Effect of enzyme usage on the extraction yield of ESM protein.

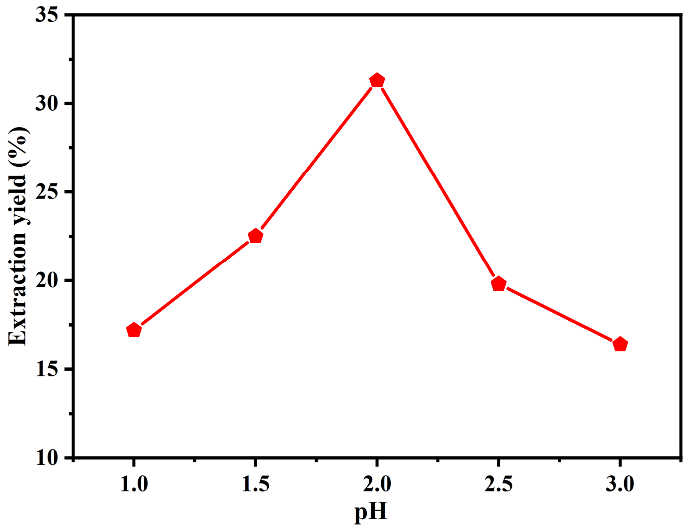

Figure S2. Effect of pH on the extraction yield of ESM protein.

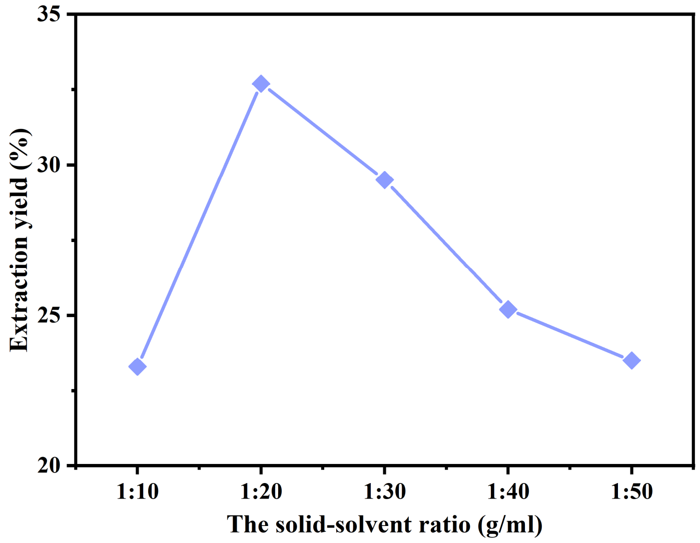

Figure S3. Effect of solid-solvent ratio on the extraction yield of ESM protein.

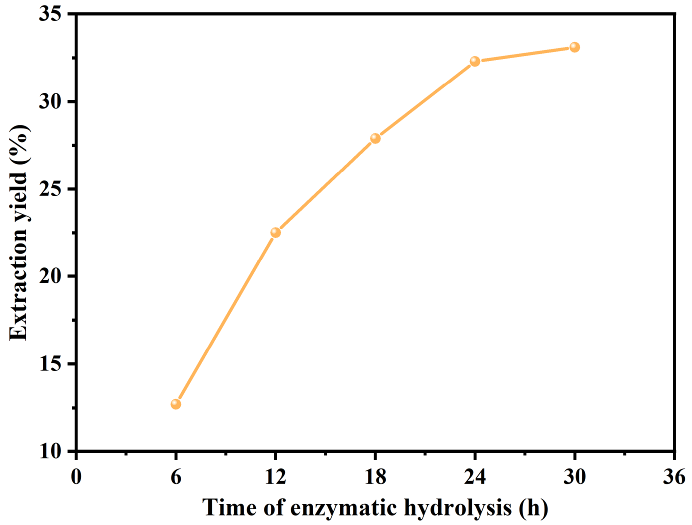

Figure S4. Effect of enzymatic hydrolysis time on the extraction yield of ESM protein.

Table S1 Box-Behnken design and results of the optimization process.

	Independent factors				Responses
Run	A	B	C	D	Extraction yield (%)
1	1% (-1)	2 (0)	1:30 (0)	30 (+1)	19.75 ± 0.31
2	3% (0)	1 (-1)	1:10 (-1)	18 (0)	20.51 ± 0.61
3	5% (+1)	2 (0)	1:50 (+1)	18 (0)	30.75 ± 0.75
4	3% (0)	2 (0)	1:30 (0)	18 (0)	30.12 ± 0.63
5	3% (0)	1 (-1)	1:30 (0)	6 (-1)	18.61 ± 0.32
6	3% (0)	3 (+1)	1:10 (-1)	18 (0)	30.42 ± 0.58
7	3% (0)	2 (0)	1:30 (0)	18 (0)	33.05 ± 0.62
8	3% (0)	2 (0)	1:30 (0)	18 (0)	34.53 ± 0.44
9	1% (-1)	1 (-1)	1:30 (0)	18 (0)	16.21 ± 0.26
10	3% (0)	2 (0)	1:30 (0)	18 (0)	35.52 ± 0.53
11	3% (0)	3 (+1)	1:50 (+1)	18 (0)	32.22 ± 0.47
12	3% (0)	2 (0)	1:30 (0)	18 (0)	34.23 ± 0.52
13	3% (0)	2 (0)	1:10 (-1)	6 (-1)	18.81 ± 0.33
14	5% (+1)	3 (+1)	1:30 (0)	18 (0)	35.21 ± 0.68
15	5% (+1)	2 (0)	1:30 (0)	6 (-1)	26.42 ± 0.56
16	1% (-1)	2 (0)	1:10 (-1)	18 (0)	18.35 ± 0.34
17	3% (0)	1 (-1)	1:30 (0)	30 (+1)	21.75 ± 0.28
18	5% (+1)	2 (0)	1:30 (0)	30 (+1)	34.22 ± 0.63
19	5% (+1)	2 (0)	1:10 (-1)	18 (0)	20.71 ± 0.36
20	3% (0)	2 (0)	1:50 (+1)	6 (-1)	19.05 ± 0.29
21	3% (0)	3 (+1)	1:30 (0)	30 (+1)	31.35 ± 0.55
22	3% (0)	1 (-1)	1:50 (+1)	18 (0)	20.33 ± 0.32
23	1% (-1)	2 (0)	1:30 (0)	6 (-1)	17.22 ± 0.25
24	1% (-1)	3 (+1)	1:30 (0)	18 (0)	30.41 ± 0.69
25	5% (+1)	1 (-1)	1:30 (0)	18 (0)	30.33 ± 0.64
26	1% (-1)	2 (0)	1:50 (+1)	18 (0)	19.21 ± 0.33
27	3% (0)	2 (0)	1:50 (+1)	30 (+1)	26.25 ± 0.51
28	3% (0)	2 (0)	1:10 (-1)	30 (+1)	25.82 ± 0.52
29	3% (0)	3 (+1)	1:30 (0)	6 (-1)	29.03 ± 0.59

Table S2 ANOVA table of the responses.

Source	Sum of square	df	Mean square	F-value	p-value*
Model	1116.94	14	79.78	16.63	<0.0001
A	265.55	1	265.55	55.36	<0.0001
B	309.07	1	309.07	64.44	<0.0001
C	14.52	1	14.52	3.03	0.1038
D	75.25	1	75.25	15.69	0.0014
AB	21.62	1	21.62	4.51	0.0420
AC	21.16	1	21.16	4.41	0.0543
AD	6.89	1	6.89	1.44	0.2506
BC	1.00	1	1	0.21	0.6550
BD	0.16	1	0.16	0.03	0.8577
CD	0.01	1	0.01	0.01	0.9642
A^2	108.90	1	108.90	22.71	0.0003
B^2	23.51	1	23.51	4.90	0.0439
C^2	244.64	1	244.64	51.00	<0.0001
D^2	189.85	1	189.85	39.58	<0.0001
Residual	67.15	14	4.80		
Lack of Fit	49.90	10	4.99	1.16	0.4823
Pure Error	17.25	4	4.31		
Cor Total	1184.09	28			
R^2	0.9433				
Adj. R^2	0.9265				
Pred. R^2	0.8862				
C.V. %	5.28				

* Significant ($p < 0.05$), Not significant ($p > 0.05$).

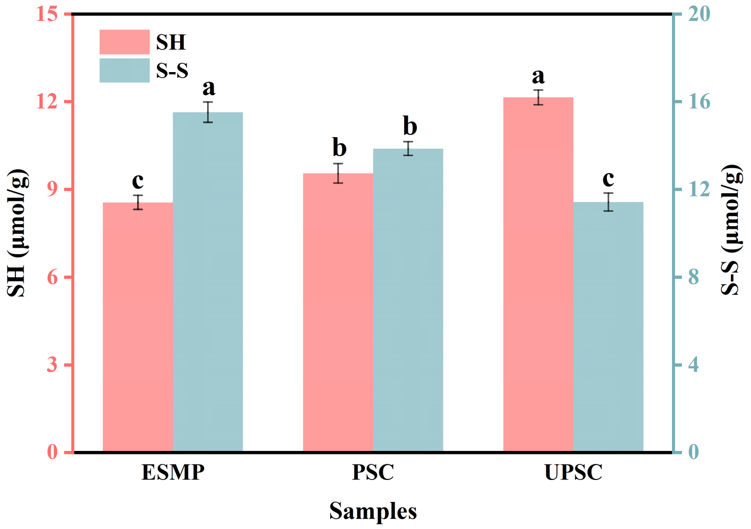

Figure S5. SH and S-S content of different samples. The different letters indicate significant differences ($P < 0.05$).

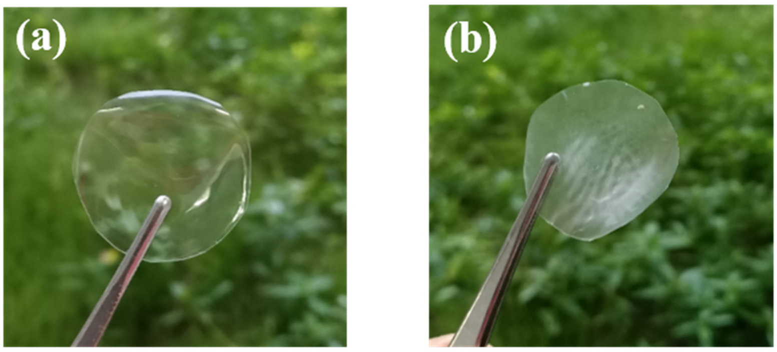

Figure S6. Photographs of (a) PVA film and (b) PVA/UPSC film, respectively.

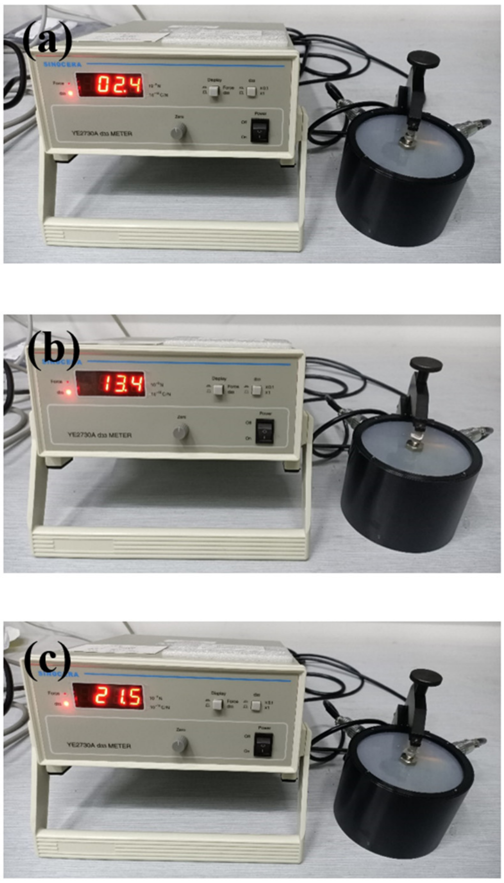

Figure S7. d_{33} of (a) ESM, (b) PVA film and (c) PVA/UPSC film measured by a commercial d_{33} meter.

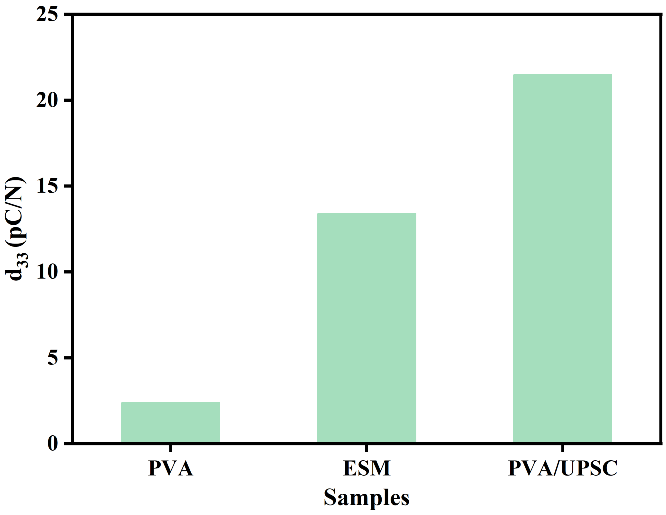

Figure S8. d_{33} of different samples.

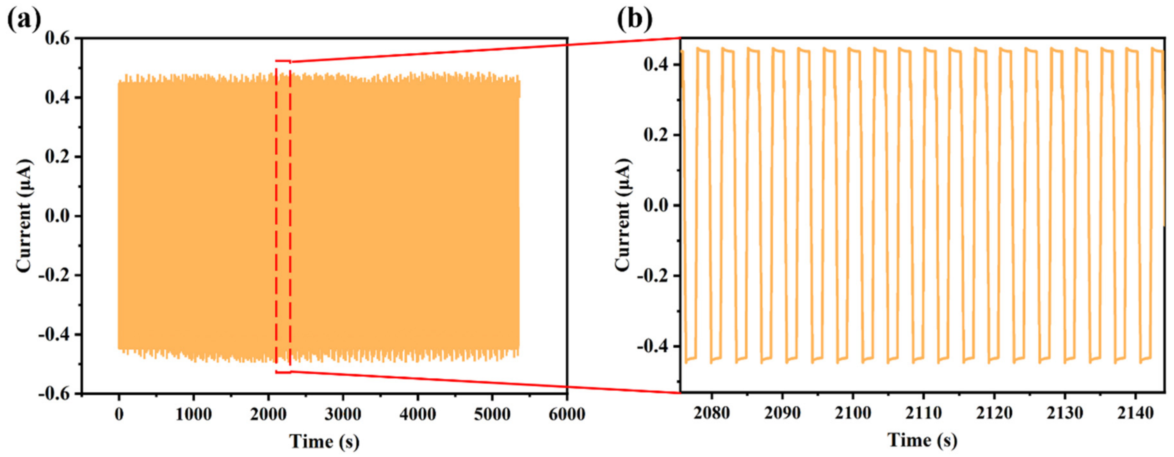

Figure S9. Stability evaluation of the device under 3000 working cycles.
